# The occurrence, types, consequences and preventability of in-hospital adverse events – a scoping review

**DOI:** 10.1186/s12913-018-3335-z

**Published:** 2018-07-04

**Authors:** René Schwendimann, Catherine Blatter, Suzanne Dhaini, Michael Simon, Dietmar Ausserhofer

**Affiliations:** 1 University Hospital Basel, Patient Safety Office, Spitalstr. 22, 4031 Basel, Switzerland; 2 Department Public Health Institute of Nursing Science, University of Basel, Basel, Switzerland; 3 American University of Beirut, School of Nursing, Beirut, Lebanon; 4 Inselspital Bern University Hospital, Nursing Research Unit, Bern, Switzerland; 5 College of Health Care-Professions Claudiana, Bozen, Italy

**Keywords:** Adverse events, Patient safety, Medical error, Hospitals, Scoping review

## Abstract

**Background:**

Adverse events (AEs) seriously affect patient safety and quality of care, and remain a pressing global issue.

This study had three objectives: (1) to describe the proportions of patients affected by in-hospital AEs; (2) to explore the types and consequences of observed AEs; and (3) to estimate the preventability of in-hospital AEs.

**Methods:**

We applied a scoping review method and concluded a comprehensive literature search in PubMed and CINAHL in May 2017 and in February 2018. Our target was retrospective medical record review studies applying the Harvard method–or similar methods using screening criteria–conducted in acute care hospital settings on adult patients (≥18 years).

**Results:**

We included a total of 25 studies conducted in 27 countries across six continents. Overall, a median of 10% patients were affected by at least one AE (range: 2.9–21.9%), with a median of 7.3% (range: 0.6–30%) of AEs being fatal. Between 34.3 and 83% of AEs were considered preventable (median: 51.2%). The three most common types of AEs reported in the included studies were operative/surgical related, medication or drug/fluid related, and healthcare-associated infections.

**Conclusions:**

Evidence regarding the occurrence of AEs confirms earlier estimates that a tenth of inpatient stays include adverse events, half of which are preventable. However, the incidence of in-hospital AEs varied considerably across studies, indicating methodological and contextual variations regarding this type of retrospective chart review across health care systems. For the future, automated methods for identifying AE using electronic health records have the potential to overcome various methodological issues and biases related to retrospective medical record review studies and to provide accurate data on their occurrence.

## Background

Adverse events (AEs) seriously affect patient safety and quality of care in hospitals. The epidemiology of harm due to medical care remains a pressing issue on a global scale. In the US, a recent report reviewing earlier studies ranked iatrogenic causes, especially medical errors, as the third leading cause of death [1]. Specifically, up to 1.1% of hospital admissions led to deaths due to medical errors. Extrapolating this percentage to the annual patient numbers for all registered US hospitals, this would account for more than 400,000 deaths in 2013 alone. While such a high volume of patient harm associated with hospital care is shocking, the projected annual cost of measurable medical errors is mind-boggling: in the US alone, based on data from that year, AEs have been dubbed “the 17.1 billion dollar problem” [2].

The two most frequent classes of AE, postoperative infections and pressure ulcers, accounted for the largest annual costs (6.5 billion USD). Following these, central venous catheter infections and infections following infusions, injections and similar procedures resulted in a combined total of more than one billion USD in extra healthcare costs.

The authors conclude that the most frequent errors result from rather common medical services for which cost-cutting efforts compromise patient safety [2]. That is, the majority of medical errors leading to in-hospital AEs are not caused by poorly performing physicians, nurses, or other clinicians. More commonly, they arise from care delivery problems that result from conditions at the levels of the individual patient or staff member, the task or the health care team. They may even be rooted in the overall work environment. If safeguards along this causal pathway fail, AEs can result. Thus, current, accurate information on AEs is crucial both for individual learning and for improving and developing more reliable health care systems [3].

Despite the existence of various national AE registries, such as the US Food and Drug Administration’s system for voluntary reporting of serious drug- or medical device-related AEs [4], or the Dutch Healthcare Inspectorate for mandatory reporting of sentinel events [5], comprehensive, prospective national-level data on in-hospital AEs are severely limited [1]. Thus, the available evidence is based mainly on retrospective chart review studies aimed at detecting AE occurrences. The two most frequently used review methods are the ‘Harvard method’ [6] and the Institute of Healthcare Improvement’s Global Trigger Tool [7]. Both involve two-stage medical record reviews and were designed to provide data on the frequency and types of AEs. Stage 1, an evaluation of the presence of certain screening criteria–triggers–is followed by a more in-depth manual review of the medical record to detect an AE (Stage 2). Table 1 provides a brief overview of the characteristics and differences between the two methods [8].

**Table 1:** Brief overview on the characteristics and differences between the ‘Harvard method’ and the ‘Global Trigger Tool method’ to detect AEs through retrospective medical record review based on Unbeck et al. [8]

Characteristics	Harvard method	Global Trigger Tool
Definition of AE	“An unintended injury or complication that results in disability at discharge, death or prolonged hospital stay and is caused by healthcare management rather than the patient’s underlying disease.”	“Unintended injury resulting from or contributed to by medical care that requires additional monitoring, treatment or hospitalization, or that results in death.”
Focus	Omission and commission	Commission, excludes omission
Method	Two - three stage retrospective record review	Two stage retrospective record review
Review Stage 1	One healthcare professional (most often nurse)	Two independent reviewers per record (e.g. nurse, physician)
Review Stage 2	Two independent reviews (most often physicians)	A team discuss the findings together Physician as arbitrator
Criterion/Trigger	- Comprehensive reading of record - Screening for one of 18 broad criteria	- No comprehensive reading - First screening for one of 54 triggers
Number of records / Time	Random, large samples	Random, small samples (e.g. 10 records every second week or 20 records every month per hospital)

The Global Trigger Tool (GTT) has the demonstrated advantage of identifying AEs more accurately than other readily available methods, including voluntary reporting of critical incidents or the Patient Safety Indicators put forth by the Agency for Healthcare Research and Quality [9]. Consequently, it has become an increasingly popular method for post hoc safety measurement and monitoring [10]. In a recent systematic review on 48 general inpatient studies using the GTT, rates of AEs varied between 7 and 40%. Of those identified, the most common event types were complications related to infections, surgical procedures and medication [11]. An earlier systematic review [12] indicated similar findings in eight studies, including a total of more than 70,000 hospital inpatient records, using the Harvard method or comparable approaches. Their analyses revealed that nearly every 10th patient was affected by an adverse event, of which 43% were deemed preventable, and 7.4% were lethal. This time, the majority of AEs were related to surgical procedures (39.6%) and medications (15.1%), while medical procedure- (7.8%), diagnostic- (7.5%) and therapeutic-related (7%) events were less frequent, they remained noteworthy [12].

Despite increasing interest among clinical practitioners and researchers to further investigate and develop the GTT, e.g., studies of the accuracy of automated methods for identifying AEs from electronic health record data [13, 14], retrospective chart review methods such as the Harvard method remain widely used to identify AEs [8]. In particular, these have been used in large-scale studies to estimate national-level AE prevalence. As a consequence, since de Vries’s 2008 systematic review on the topic, numerous retrospective chart review studies have applied Harvard-style methodologies [9].

## Methods

### Aim

The purpose of this study was to update recent in-hospital adverse event figures according to three objectives: (1) to describe the proportions of patients affected by in-hospital AEs; (2) to report the types and consequences of observed AEs; and (3) to estimate the preventability of in-hospital AEs.

### Design

We conducted a scoping review based on the framework outlined by Arksey and O’Malley [15]. A scoping review’s purpose is to summarize “a range of evidence in order to convey the breadth and depth of a field” [16]. It differs from a systematic literature review in that it requires broader research aims (as opposed to a narrowly focused question) and more refined post hoc (rather than a priori) selection criteria for papers during the review process, but suspends critical appraisal of bias risk or other quality indicators until after the selection process is complete [15, 17–19].

### Literature search

**Fig. 1:**
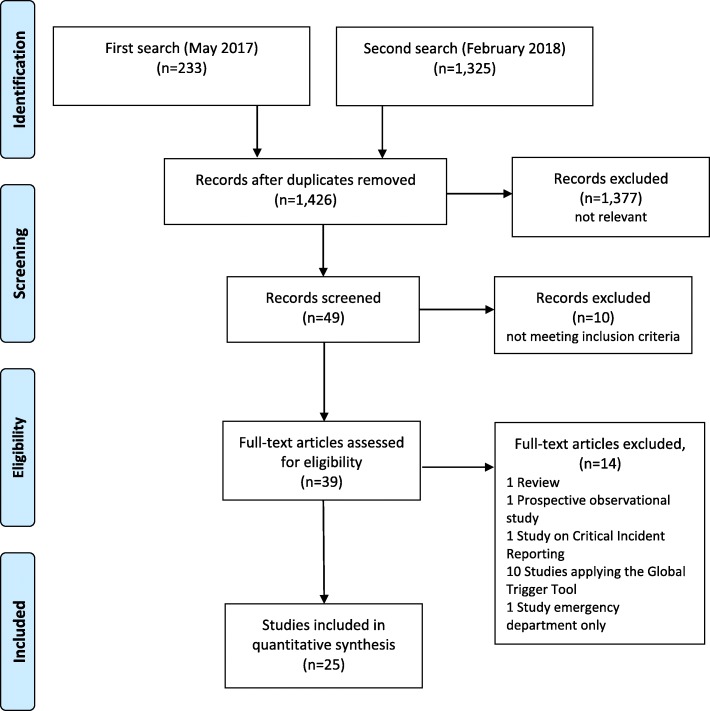
Flow diagram of article retrieval

A first literature search of the Medline (PubMed) and CINAHL databases was conducted in May 2017 using the terms “*Incidence” [tiab] AND “adverse events” [tiab] AND “patient safety.”* The records retrieved were screened independently by CB and SD for hospital adverse event studies conducted between 1991 and 2017. A manual search of the returned articles’ bibliographies was also performed to identify additional relevant studies. In February 2018, in line with our scoping review methodology, a second comprehensive database search was conducted to confirm and complement the results of the first PubMed literature search using an elaborate search string using Hausner et al.’s approach: ((adverse [tiab] AND events [tiab] AND patient*[tiab]) OR incidents [tiab]) AND preventable [tiab])) [20]. Following Hausner et al.’s model, we developed our search string based on a primary set of relevant records (a development set) identified in the first search. The relevant records’ titles and abstracts contained keywords (e.g., MeSH terms) and free text terms that served as the building blocks of the final search string. Variations of the search string were then tested against a second set of relevant records (validation set). In our case, as no MeSH term for “adverse events” existed and no promising alternative keywords could be identified, a free text only search string was compiled. All records retrieved were screened independently by RS and DA (see Fig. 1).

### Study selection

In order to reliably compare the data, we defined an AE according to the Harvard Medical Practice Study definition: “an unintended injury or complication that results in disability at the time of discharge, death or prolonged hospital stay caused by health care management rather than by the patient’s underlying disease process” [21]. All primary studies that (1) used this or a similar definition of an AE, (2) applied a retrospective medical record review according to the Harvard method or a similar method, (3) used a two-stage record review with primary criteria-driven screening by trained reviewers (mostly nurses) and a secondary review of their findings by a physician, (4) were conducted in acute care hospital settings (e.g., medical, surgical, ICU), and (5) included hospitalized adult patients (≥18 years), were eligible for inclusion. Studies restricted to specific clinical areas (e.g., pediatrics, emergency department), studies from non-acute care settings (e.g., rehabilitation, long-term care, primary care), as well as those that evaluated only specific types of AE (e.g., adverse drug events), were excluded. Systematic reviews or studies applying prospective methods (e.g., critical incident reporting systems) were also excluded. Likewise, given that differences in review methodologies result in disparities in AE detection [8], and that a systematic literature review on the application of the GTT had recently been published [11], studies applying the GTT method were excluded. Final inclusion of studies was achieved by consensus between the authors (RS and DA).

### Data charting and analysis

From the selected studies, we extracted information on the prevalence (i.e., the proportion of affected patients), types, frequencies, and related consequences of AEs, those AEs’ preventability, and relevant study details (i.e., methods, number of hospitals, setting and sample size) (see Table). Critical appraisal of the included studies and meta-analysis of AE rates across studies were not undertaken due to methodological heterogeneity (e.g., differences in setting, retrospective chart review methods, number of screening criteria, number of reviewers, detection and inclusion of AEs before index admission). Medians and ranges of occurrence, types, consequences and preventability of AEs were calculated using Microsoft Office Excel 2016.

## Results

### Study selection

We included 25 studies conducted between 1991 and 2017 in 27 countries in Europe, Asia, Africa, North and South America, and Australia/New Zealand (see Table 2). All included studies used two-stage retrospective medical record reviews to detect in-hospital AEs based on the ‘Harvard method’ [6] or modified versions of it, such as those developed in Canada [22] and the United Kingdom [23]. Of the 25 studies used, 19 were multi- and 6 single-centre studies. In the multicentre studies, the number of hospitals ranged from two to 58, with sample sizes ranging from 354 [24] to 30,121 [6] patients/records. The single-centre studies’ samples ranged from 500 [25] to 1501 [26] patients/records (see Table 2).

**Table 2:** Frequencies of occurrence, characteristics/types, consequences and preventability reported in the included studies on in-hospital AEs

Authors (publication year), Country	Hospital/ Setting/ Sample size	Occurrence of AEs	Characteristics/Types of AEs	Consequences of AEs	Preventable AEs
*Name of the first author (Publication year), Country/countries*	*Number of study sites; setting of the study (*e.g. *medical, surgical, ICU) (number of patients / medical records included in the study)*	*Frequency of AEs observed in the study*	*Frequency/Distribution of the characteristics/types of AEs*	*Consequences of AEs according to classification used in the study (*e.g.*, temporary or permanent harm, death,* etc.*)*	*Percentage of AEs judged as preventable*
Akbari et al. (2015), Iran, [34]	4 general hospitals; medicine, general surgery, urology, orthopaedics, ICU, CCU, A&E, ENT, ophthalmology, paediatrics and women’s health (*n* = 1162),	7.3% during stay (3.7% of patients had AEs before admission)	Adverse drug reaction (26.6%); post-op infections (19.5%); pressure ulcers (13.3%); hospital-acquired infections (10.2%); procedural complications (10.2%)	Minimal impairment (< 1 month): 73.4%; moderate impairment (1–12 months): 21.2%; severe impairment: 3.9%; death: 0.8%	34.3%
Aranaz-Andres et al. (2011), Argentina, Colombia, Costa Rica, Mexico, Peru, [51]	58 hospitals; surgery, gynaecology, obstetrics, paediatrics and intensive care (*n* = 11,379)	10.5% (1191 patients) had at least one AE	Hospital-acquired infections (37.1%); surgical procedures (28.5%); patient management and nursing care (13.4%); medication (8.2%); diagnostics (6.1%). The 5 most frequent AEs (accounting for 36.2% of all AEs): 127 hospital-acquired pneumonia (9.4%); 111 surgical wound infections (8.2%); 97 pressure ulcers (7.2%); 86 other complications related to surgery or procedure (6.4%) and 67 sepsis (5%).	Any kind of disability: (28.8%); death (5.8%)	59%
Aranaz-Andres et al. (2009), Spain, [32]	24 hospitals, all wards (*n* = 5624)	9.3% (525 patients; 655 AEs) had at least one AE	Medication use (37.4%); nosocomial infections (25.3%); procedure-related (25%); care-related (7.6%); diagnosis-related (2.7%); others (1.8%)	Minor: (45%); moderate: (39%) and severe: (16%resulted in a longer stay: (31.4%); death (4.4%)	42.6% (overall) (84.2% of all diagnostic AEs, 56.6.% of nosocomial infections, 56% care related AEs)
Baker et al. (2004), Canada, [22]	20 hospitals; medical-surgical units (*n* = 3745)	7.5% (255 patients; 289 AEs) had at least one AE	Surgical procedures (34%), drug-related events (24%), clinical management (12%), diagnostic procedures (11%), medical procedures (7%), others e.g., burns, falls (5%)	Minimal impairment (< 1 month): 64.4%; moderate impairment (1–12 months): 12.5%; permanent impairment: 5.2%; death: 15.9%	36.9%
Brennan et al. (1991), US, [6]	51 hospitals; acute care (*n* = 30,121)	3.7% (1278 AEs)	Not stated	Minimal impairment (= < 1 month): 56.8%; moderate impairment (> 1 month): 16.5%; permanent disability: 6.5%; death: 13.6%	Preventability not stated;27.6% AE due to negligence
D’Amour et al. (2014), Canada, [52]	11 hospitals; medical units (*n* = 2699)	15.3% (412 patients; 568 AEs) had at least one AE	Falls (40.5%); administration errors (29.7%); urinary tract infection (12.3%); pressure ulcers (9.1%); inappropriate use of restraints (4.2%); medication pneumonia (4.0%)	167 of AE (29.4%) had consequences	Preventability not stated; 76.8% AEs due to inappropriate nursing care
Davis et al. (2002) & Davis et al. (2003), New Zealand [53, 54]	13 hospitals; acute care (*n* = 6579)	12.9% (850 AEs)	Operative (24.3%), system: (24%), drug (12.3%), therapy (8.4%), diagnosis (8%), procedure (7.7%), other (e.g., falls) (15.3%)	Minimal impairment (< 1 month): 61.6%; moderate impairment (1-12 months): 19.0%, permanent disability: 10.2%; death: 4.5%; unclear (4.7%)	37.1%
Forster et al. (2004), Canada, [35]	1 hospital (multi-campus); (*n* = 502)	12.7% (64 patients)	Adverse drug events (50%), surgical complications (31%), nosocomial infections (19%), diagnostic errors (9%), system problems (8%), procedure injuries (8%), anaesthetic injuries (2%), obstetric injuries (2%)	Temporary disability: 10.4%; permanent disability: 1.8%; death (not preventable): 0.6%	37.5%
Grira et al. (2015), Tunisia, [25]	1 hospital; internal medicine (*n* = 500)	5.2% (26 patients)	Adverse drug events (73%), healthcare associated infections (19%), non-surgical procedures (4%), pressure ulcers (4%)	Prolongation of hospitalization: (27%); disability: (15.4%); readmission: (42.9% of hospitalizations due to AE)	57.7%
Halfon et al. (2017), Switzerland, [55]	1 medium size community hospital; medical and surgical setting (*n* = 1007)	12.3% AEs (64 AEs in 55 medical patients, 82 AEs in 72 surgical patients)	Surgical patients: operative procedures (74%), medications (15%), non-surgical procedures (7%), diagnostic procedures (4%), medical patients: medication (52%), non-surgical procedures (27%), operative procedures (11%), therapeutic decisions (6%), diagnostic procedures (5%)	No or minimal impairment: 60%, Severe impairment: 23%	42%
Kable et al. (2002), Australia, [28]	28 hospitals; surgery (*n* = 5432)	21.9% (1190 AEs)	Operation-related (74.9%): wound infections (2.1%); bleeding (1.4%), wound problems (1.2%), deep vein thrombosis/pulmonary embolisms (0.3%), and pneumonia (0.2%)	Minimal disability (< 1 month): 46.9%, moderate impairment (1-12 months): 36.1%,permanent disability: 17%; death: 4.0%	47.6% (highly preventable)
Letaief et al. (2010), Tunisia, [33]	1 hospital; 18 units (*n* = 620)	10% (62 AEs)	Surgical/invasive related (54.8%), therapeutic errors (20.9%), diagnostic errors (12.9%), drug-related (6.5%); others (4.9%)	Minimal impairment (< 1 month): 16.1%moderate impairment (1–12 months):56.5%permanent disability: 6.4%; death: 21%	60%
Mendes et al. (2009), Brazil, [56]	3 hospitals; acute care (*n* = 1103)	7.6% had at lease one AE (84 patients; 103 AEs)	Surgical procedures (35.2%), medical procedures(30.6%), diagnoses (10.2%), obstetric (8.3%), medication (5.6%,), fractures (1.9%), anaesthetic (0.9%), system events (6.5%), others (0.9%)	Not stated	66.7%
Rafter et al. (2017), Ireland, [57]	8 hospitals; acute care (*n* = 1574)	12.2% (247 AEs)	Operation related (25.48%), therapeutic events (24.55%), medication related (14.1%), diagnostic events (11.55%), other events, not covered elsewhere (9.25%), non surgical procedure related (7.9%), fracture related (3.92), pregnancy related (1.17%), anaesthetic related (1.14%), fluid related (0.94%)	Minimal impairment (< 1 month): 33.6%moderate impairment (1–12 months): 25.8% permanent disability: 9.9%; death: 6.7%	72.7%
Sari et al. (2007), England, [29]	1 hospital (*n* = 1006)	13.5% (136 AEs)	Not stated	Not stated	Not stated
Sommella et al. (2014), Italy, [26]	1 hospital (*n* = 1501)	3.3% (46 AEs)	Not stated	Not stated	Not stated
Soop et al. (2009), Sweden, [58]	28 hospitals; acute care units (*n* = 1967)	12.3% (241 AEs)	Invasive procedures including surgical operations (49.4%), drug treatment (30.1%), diagnostic procedure (11.3%), other procedures (14.2%)	Minimal impairment (< 1 months): 53.5% moderate impairment (1–12 months): 29.8% permanent disability: 10.8%; death: 4.1%	70.1%
Sousa et al. (2014), Portugual, [30]	3 hospitals; acute care (*n* = 1669)	11.1% (186 AEs)	Surgical related (27%), drug errors (18.3%), hospital acquired infections (12.2%)	Minimal impairment (< 1 months): 61% moderate impairment (1–12 months): 4.1% permanent disability: 5.7%; death: 10.8%	53.2%
Tartaglia (2012), Italy, [59]	5 hospitals (*n* = 7573)	5.2% (*n* = 470 Aes in 386 patients)	Medical patients (37.5%), surgical patients (30.1%), emergency department (6.2%), obstetric patients (4.4%),	Prolongation of hospitalization (66.7%); disability (18.0%); death (10.6%)	56.7%
Thomas et al. (2000), US, [27]	28 hospitals; all acute care units (*n* = 14,700)	2.9% (587 AEs)	Surgery (44.9%), drugs (19.3%), medical procedures (13.5%), diagnoses (6.9%), therapy (4. 3%), obstetric (3.6%), falls (1.3%), fractures (0.4%), others (1.5%	Temporary impairment: 73.8%,permanent impairment: 7.5%; death: 6.6%	Preventability not stated;29.2% negligent AEs
Vincent et al. (2001), Great Britain, [23]	2 hospitals; general medicine, general surgery, orthopaedics, obstetrics (*n* = 1014)	10.8% (110 patients, 119 AEs) with at least one AEs	General medicine (9.2%), general surgery (16.2%), obstetrics (4%), orthopaedics (14.4%)	Minimal impairment: 66% moderate impairment: 19%permanent disability: 6%; death: 8%	48%
Williams et al. (2008), Scottland, [24]	2 hospitals, acute medical, surgical and obstetric admissions (*n* = 354)	7.9% (Range: 0% obstetrics, 7.2% medicine, 13% surgery)	Nature of problem: medical and nursing management and monitoring (32.1%), infection related (35.7%), technical procedure related (21.4%), drug/i.v. fluid problem (7.1%), fall (3.6%)	No physical impairment (17.9%), minimal physical impairment (35.7%), moderate impairment (28.6%), permanent impairment (7.1%), contributed to patient death (10.7%)	43%
Wilson et al. (2012), Egypt, Jordan; Kenya; Morocco; Tunisia; Sudan; South Africa; Yemen; [31]	26 hospitals; paediatric hospital; obstetric hospital; general public hospital; teaching hospital (n = 15,548)	8.2% (between country-variability ranging from 2.5 to 18.4%)	Therapeutic errors (34%), diagnostic (18%), operative (17%); obstetrics (8%), neonatal (7%), non-surgical procedures (5%),drug related (4%), fractures (3%), falls (2%), anaesthesia (2%)	Minimal impairment (< 1 months): 32% moderate impairment (1–12 months): 16% permanent disability: 12%; death: 30%	83%
Wilson et al. (1995), Australia, [60]	28 hospitals (n = 14,179)	16.6%	Operative (50.3%), diagnoses (13.6%), therapy (12%), drug (10.8%), medical procedures (8.6%), fractures (5.5%), obstetrics (5.5%), falls (2.9%), others (19.1%)	Minimal disability: 46.6% permanent disability: 13.7%; death: 4.9%	51.2%
Zegers et al. (2009), Netherland, [61]	21 (university, teaching and general) hospital (*n* = 7926),	5.7% (6.8% surgical; 4.8% non-surgical)	Surgery (54.2%), medical procedures (17%), drug/fluid (15.3%), diagnostic (6.3%), other clinical management (3.7%), other (e.g. falls) (2.1%), discharge (1.4%)	No or minimal physical impairment: 56.8%permanent disability: 5.0%; death: 7.8%	39.6% (surgical: 39.5%, non-surgical 40.3%)

### Frequencies of AEs

Across all studies, the frequencies of patients with AEs spanned between 2.9% [27] and 21.9% [28]. The overall median was 10%, with multicentre studies showing a median of 9.3% (range: 2.9% [27] – 21.9% [28]). Single centre studies reported a median of 11.2% with an overall range of 3.3% [26] to 13.5% [29] patients affected by AEs.

### Types of AEs

The most common and most consistently reported types of AE in the included studies were operative/surgical-related events, often resulting from procedural complications and injuries such as post-op bleeding or return to surgery. These accounted for a median of 40% of those detected (range: 27% [30] – 74.9% [28]). The second most frequent type was medication- or drug/fluid-related events such as medication errors, which accounted for a median of 19.3% of those detected (Range: 4% [31] – 73% [25]). In third position, healthcare-associated infections and allergic reactions were responsible for a median of 17.7% of all detected events (Range: 0.2% [28] – 25.3% [32]).

### Severity and preventability of AEs

AEs’ consequences were mostly temporary and minimal (recuperation < 1 month) (Median: 53.5%; Range: 16.1% [33] – 73.4% [34]) or caused no patient harm. However, 21.2% of those affected suffered from moderate impairment (recuperation 1–12 months) (Range: 4.1% [30] – 56.5% [33]). A median of 7.3% of AEs resulted in permanent disability (< 50% and > 50% disability) (Range: 3.9% [34] – 17% [28]). Death occurred in a median of 7.3% of patients affected by at least one AE (Range: 0.6% [35] – 30% [31]). Across all included studies, a median of 51.2% of events were considered preventable (Range: 34.3% [34] – 83% [31]). In the reviewed studies, an AE was classified as preventable based on the chart reviewer’s professional judgement of the given incident and consent. In most of the selected studies, preventability of AEs was determined based on a 6-point scale range from “virtually no evidence of preventability” (1 point) to “virtually certain evidence of preventability” (6 points), with a cut-off score of 4.

## Discussion

We conducted a scoping review and provided an updated international overview of studies on the prevalence, characteristics/types, consequences and preventability of reported hospital AEs. Included studies that applied a retrospective chart review methodology based on the ‘Harvard Medical Practice Study’ approach or similar methods published after 2008 confirmed the findings of de Vries et al.’s systematic review on the worldwide magnitude of in-hospital AEs [12]: one out of ten hospitalized patients is affected by at least one AE, with one out of 14 such events resulting in fatality and half of all cases considered preventable. These findings on AEs’ occurrence are comparable to those reported in studies either applying the Global Trigger Tool methodology [11] or relying on patients’ reports of having experienced medical errors [36]. Applying these figures to the Swiss context in 2015 [37] would mean that roughly 140,500 in-patients in Swiss hospitals experienced AEs, resulting in about 9′400 fatalities.

Although we included studies that used similar definitions for AEs and retrospective record review methodologies similar to the Harvard Medical Practice Study [38], the occurrence of in-hospital AEs varied considerably across studies. A number of methodological differences including setting/sample (e.g., medical and/or surgical patients), inclusion of events before or after index admission, the number and types of screening criteria, thresholds for defining causation and preventability or the number, professional background, and the chart reviewers’ level of experience [39, 40], may partly account for the variability observed in in-hospital AEs across countries. It must be acknowledged that retrospective record review methodology is at risk of bias, including hindsight and performance bias, which would lead respectively to over- or underestimation. However, reducing methodological heterogeneity is crucial to achieving accurate comparisons between countries and meta-analyses of AE rates across studies. The development of a reporting guideline on retrospective chart review studies, e.g., based on the “Strengthening the Reporting of Observational Studies in Epidemiology” (STROBE) statement [41], could contribute to standardization of the performance and reporting of AE studies.

Moreover, contextual factors within healthcare systems, such as variation in the quality and methods of medical and patient record documentation across countries and hospitals, might be a key source of variation in AE detection. In combination with the GTT, electronic health records (EHR) are currently gaining the interest of researchers and clinical practitioners alike, as they offer tremendous opportunities to develop automated AE identification methods [14]. While such an approach appears to be time-saving and less resource intensive compared to manual retrospective record review, it demands consistent documentation and representation of key EHR data elements. However, using machine learning and natural language processing in electronic health records, the possibilities to detect and monitor AEs are expanding rapidly. The most advanced systems are already providing real-time feedback to healthcare professionals, thereby offering hospitals advanced quality improvement and learning opportunities [42]. While the results obtained via EHR analyses are surprisingly similar to those available via manual review, computerized searches are virtually instantaneous and cost very little after the initial infrastructure is in place.

Despite variations between in-hospital AE incidence and measurement, our findings confirm both AEs’ harmful impacts on patients and the persistent need for effective preventive measures. As noted above, the three main types of AEs reported in the included studies were related to surgery, medication/drugs and healthcare-associated infections. In recent years, the study of quality improvement interventions has led to major progress in patient safety [43], with evidence on effective strategies readily available [44]. For example, hospitals can adopt individual or bundled interventions. Adapted from aviation industry methodology, these are aimed at reducing the three major types of AEs, and include the use of checklists in the operating room [45], care bundles for the insertion of central venous catheters [46, 47], hand hygiene adherence [48], and medication reconciliation practices [49]. Acute care hospitals need to utilize comprehensive and balanced frameworks such as those available to measure, monitor [50] and improve care safety, as well as to foster a culture of safety, especially concerning the three main AE types. Accurate monitoring of in-hospital AEs, including via retrospective record reviews, is essential for the implementation and evaluation of evidence-based strategies to reduce their occurrence and patient harm.

### Limitations

This scoping review should be read in view of certain limitations. First, we conducted our literature search in the two major electronic databases, Medline (Pubmed) and CINAHL, but did not search for “gray” literature. Therefore, additional relevant studies might have been missed. Second, in line with the scoping review methodology, we did not assess the included studies’ risk of bias. Although we observed methodological heterogeneity in the included studies we did not include/exclude studies based on a quality assessment, as would be necessary in a systematic review. Therefore, caution is advised when drawing conclusions based on these studies’ combined data.

## Conclusions

This scoping review included 25 studies conducted in 27 countries. All had applied the ‘Harvard Method’ of detecting AEs. The median overall occurrence was 10%. Of this number, half were regarded as preventable and 7.3% led to fatal outcomes. However, the occurrence of in-hospital AEs varied considerably across studies, indicating methodological variation and cultural/contextual differences in conducting this type of retrospective chart review. Further research, such as on automated methods of identifying AEs in electronic health records, is needed to overcome methodological issues and bias related to this type of retrospective medical record review and to provide accurate data on their occurrence. As the three most common and most consistently reported types of in-hospital AEs were related to surgery, medication and nosocomial infections, further efforts to measure and monitor these three areas will make hospital care safer and more reliable.

## Abbreviations

AE: Adverse events
EHR: Electronic Health Record
GTT: Global Trigger Tool
ICU: Intensive care Unit
MeSH: Medical Subject Headings
